# Days out of role due to mental and physical illness in the South African stress and health study

**DOI:** 10.1007/s00127-014-0941-x

**Published:** 2014-08-06

**Authors:** Sumaya Mall, Crick Lund, Gemma Vilagut, Jordi Alonso, David R. Williams, Dan J. Stein

**Affiliations:** 1 Alan J Flisher Centre for Public Mental Health, Department of Psychiatry and Mental Health, University of Cape Town, Cape Town, South Africa; 2 Health Services Research Unit, IMIM- Institut Hospital del Mar d’Investigacions Mediques, Barcelona, Spain; 3 CIBER Epidemiologia y Salud Publica (CIBERESP), Barcelona, Spain; 4 Universitat Pompeu Fabra, Barcelona, Spain; 5 Department of Social and Behavioural Sciences, Harvard University, Cambridge, USA; 6 Department of Psychiatry and Mental Health, University of Cape Town, Cape Town, South Africa

**Keywords:** Mental disorders, Physical disorders, South Africa, Days out of role, SASH

## Abstract

**Background:**

Both mental and physical disorders can result in role limitation, such as ‘days out of role’, which have an important impact on national productivity losses. This paper analyses data from the South African Stress and Health Study (SASH) on the association of both mental and physical disorders with days out of role.

**Methods:**

Face-to-face interviews were conducted with a representative sample of 4,351 adult South Africans. The World Health Organization’s Composite International Diagnostic Interview (WHO-CIDI) was used to assess the presence of 21 mental and physical disorders that were grouped into 10 disorder categories for the analysis: major depressive disorder, any anxiety disorders, any substance abuse disorders, headaches or migraine, arthritis, chronic pain, cardiovascular, respiratory, diabetes and digestive disorders. Multiple regression techniques were used to explore associations between individual disorders, comorbid conditions, and annual days spent out of role. The estimated societal effects of the disorders [population attributable risk proportion (PARP)] were obtained.

**Results:**

The majority of respondents who reported a mental or physical disorder also reported another disorder (62.98 %). The average number of disorders reported by respondents who had at least one disorder was 2.3. Overall 12.4 % of respondents reported any days out of role due to mental or physical disorder. Anxiety disorders and depression were associated with highest days out of role (28.2 and 27.2, respectively) followed closely by arthritis and pain (24.7 and 21.7, respectively). Any mental disorder was associated with 23.6 days out of role, while any physical disorder was associated with 15.5 days out of role. Of the mental disorders, anxiety disorders had the highest PARP in relation to days out of role (9.0 %) followed by depression (4.8 %) and substance disorder (3.3. %). More than one-third (37.6 %) of days out of role are attributable to physical disorders and 16.1 % to mental disorders.

**Conclusions:**

Comorbidity is common in both mental and physical disorders, and both are associated with substantial days out of role in South Africa. These data indicate substantial social and economic loss associated with these conditions, and emphasize the need to integrate health services to include common mental disorders in all basic packages of care and to assess for and manage comorbid conditions.

## Introduction

Mental and physical disorders, which are highly prevalent both globally and in South Africa have disabling consequences [1–3]. These include ‘days spent out of role’, a measure of inability to work or carry out day-to-day activities. ‘Days out of role’ has been widely used as a measure of disability [4]. In the United States of America (USA), studies have estimated that 3.6 billion annual health related days out of role can be attributed to mental disorders [5]. In Europe, annual costs of productivity losses from days out of role due to brain disorders exceed €178 billion [6].

Research on the impact of different illnesses on both partial and full disability is emerging [7–9]. The USA National Comorbidity Replication Survey (NCS-R) found that mental disorders in the USA are widespread and affect those with comorbid conditions i.e. having more than one disorder occurring together with the primary condition [10]. There has been research exploring the relationship between full disability (i.e. the number of days totally unable to carry out normal activities due to health problems) and partial disability (i.e. the number of days where individuals are partially unable to perform as usual) and specific disorders [11, 12]. For example, Bruffaerts et al. [11], report that their respondents with mental disorders from 26 nationally representative samples reported 15–28 % more partial disability days than respondents with physical disorders. Relatively little research has adjusted for high rates of comorbidity between physical and mental disorders [12]. The World Mental Health Surveys (WMHS) [12] have provided some of the first data that do so. However, there is little detailed data available from low and middle-income countries such as South Africa.

The South African Stress and Health Study (SASH), the country’s first nationally representative survey of mental disorders, offers an opportunity to fill this gap. The SASH survey, like others in the WMHS, provides detailed data on a range of physical and mental disorders, as well as on days out of role. These data allow researchers to obtain detailed odds ratios (OR) of the association between different comorbid conditions and estimates of the association between physical and mental disorders and days out of role. The data provide a unique opportunity to calculate days out of role associated with both physical and mental disorders in the South African context.

In this paper we aim to obtain estimates of the relationship between days out of role and comorbid physical and mental health conditions in the South African context, and offer directions for policy makers in designing interventions that address comorbid physical and mental conditions. To date this is the only nationally representative survey of psychiatric disorders on the African continent.

## Methods

### Sample characteristics

The sample characteristics of the South African Stress and Health Study have previously been reported [13–15]. The primary aim of the survey was to determine prevalence of mental disorders in a nationally representative sample of 4,351 adult (i.e. 18 years and older) South Africans living in both households and hostel quarters, a common place of residence for South Africa’s migrant labourers. Individuals of all racial and ethnic backgrounds were included in the study to ensure representativeness of the South African population.

### Sampling procedure

The sample was selected using a three-stage clustered area probability sample design. The first stage involved the selection of stratified primary sample areas based on the 2001 South African Census enumeration areas (EAs). The second stage involved the sampling of housing units within clusters selected within each of these EAs. Sampled residences were stratified into 10 diverse housing categories: rural–commercial, agricultural, rural traditional subsistence areas, African townships, informal urban or peri-urban shack areas, Coloured^1^ townships, Indian townships, general metropolitan, residential areas, general large metropolitan residential areas, and domestic worker accommodation in urban areas. Within each of these strata, 600 households were derived from maps, census data, or aerial photographs. The third stage involved the random selection of one adult respondent in each sampled housing unit. Fieldwork supervisors had the task of selecting the households and the respondents. The supervisors visited each (EA), counted the residences and randomly selected the sample houses for that EA. The supervisors then assisted the interviewers with recruiting the residents at that dwelling and selecting the sampled individual using the Kish method of probability (based on a comprehensive, full list of all members of the household by age and gender). At this stage informed consent was obtained and a time was scheduled for the interview [15]. The data were collected between 2002 and 2004. Face-to-face interviews took place in all the South African provinces. The overall response rate was 86 %. Interviews lasted approximately 3–4 h and were conducted in several South African languages.

### Measurement

The measuring instrument employed in the SASH study is the paper and pencil version of the World Health Organization’s Composite International Diagnostic Interview CIDI) version 3.0. The instrument is designed for use by non-clinical interviewers and allows diagnoses of mental disorders according to the definitions and criteria of both the International Classification of Diseases (ICD) and Diagnostic and Statistical Manual of Mental Disorders (DSM) systems, although only DSM-IV criteria are used in this case. Fieldworkers were trained to use the measuring instrument. The fieldworkers were only selected for interviewing if they demonstrated that they were competent to use the measuring instrument. The measuring instrument was translated and back translated with advice from language experts who were familiar with WHO translation recommendations. Generally good concordance was found between mental disorders diagnoses based on the CIDI and clinical diagnoses from blinded clinical re-interviews [16] using the Structured Clinical Interview for DSM-IV (SCID) [17] as the gold standard. Areas under the receiver operator characteristic curve (AUC) were in the range 0.7–0.8. With regard to physical conditions, we focused on commonly occurring chronic physical conditions that have been shown in previous research to be associated with significant role impairments [5, 18]. Physical disorders were self-reported using a checklist for chronic disorders, a procedure employed in previous studies exploring the relationship between chronic and physical conditions [16–18].

A total of 21 physical and mental disorders were combined into 10 disorder categories to be used as predictor variables in this analysis: major depressive disorder, any anxiety disorders [panic disorder and/or agoraphobia, social phobia, generalized anxiety disorder or post-traumatic stress disorder (PTSD)], any substance abuse disorders (alcohol abuse with and without dependence or drug abuse with and without dependence), headaches/migraine, arthritis, pain (chronic back or neck pain or other chronic pain), cardiovascular (heart attack, heart disease, hypertension or stroke), respiratory (seasonal allergies, asthma, chronic obstructive pulmonary disease or emphysema), diabetes and digestive disorders (stomach or intestinal ulcers or irritable bowel disorder). As in the World Mental Health survey [12], we focus on disorders present during the year prior to the interview. The chronic conditions were grouped into seven categories to maximize comparability with the categories used in the WHO Global Burden of Disease study [19]. Also, due to low prevalence, the five anxiety disorders were grouped into a single category, as were alcohol and drug disorders.

### Days out of role

To obtain data of days out of role, the SASH survey incorporated a modified version of the WHO Disability Assessment Schedule version 2.0 (WHODAS), an instrument that has been widely used in population-based studies [20–22]. The WHODAS assesses disability under several different domains including general role impairment, mobility, ability to care for oneself and general social and cognitive functioning [22]. This was employed to ask survey respondents the number of days (beginning on the day before their interview and going back 30 days prior to the interview) that they felt they were totally unable to work or carry out their day-to-day functioning due to problems with their mental or physical health or drugs. The validity of self-report of days out of role has been well established by studies comparing pay roll records of employed people with self-reports of days out of role [23–25].

### Analysis

Multiple regression analysis techniques were used to examine the associations of several predictor variables (i.e. the 10 physical and mental disorder categories assessed in the survey) with the dependent continuous variable (i.e. reported days totally out of role during the past 30 days). The model controlled for a series of socio-demographic variables which are thought to have substantial bearing on days spent out of role including age, sex, employment status, and education. As there was substantial comorbidity in the sample, additional terms were included in the model for total number of comorbid disorders and separate interaction terms between the type and number of comorbid disorders, to account for the possibility that the effect of a given disorder on days out of role varies depending on the presence or absence of other disorders. As the outcome variable (a 0–30 measure of number of days out of role) was highly skewed (a considerable proportion of individuals had no disability) special modelling procedures were used to address the fact that ordinary least square regression methods may yield biased and inefficient results when these kind of outcomes are predicted. To do this, seven different model specifications were compared through standard procedures of model fit [26]. The specifications included an ordinary least squares regression model and six different generalized linear models (GLM) consisting on the combinations of two link functions (logarithmic or square root) and three error structures (constant variance, variance proportional to the mean and variance proportional to the mean squared). The best-fitting specification was found to be a GLM model with logarithmic link function and variance proportional to the mean (detailed results of model comparison are available under request).

As the regression model includes the interaction terms, the predictive effect of each disorder is distributed across a number of different coefficients. Simulation was then used to produce a single term to summarize all these components effects, denominated as average individual-level effect. This was done by estimating the predicted value of the outcome for each respondent from the coefficients in the final model (the base estimate) and a disorder-specific simulated predicted estimate also applying the same coefficients but assuming that one particular disorder was no longer present (i.e., setting at zero those with the disorder). This exercise was repeated 10 times (i.e., one for each different disorder category considered). As a result, 10 pairs of estimates (a base and a simulated estimate for each disorder category) were obtained for all individuals. The differences within each pair (base estimated minus simulated estimate) represented the additional days out of role of each respondent associated with each of the 10 particular disorders. These differences were averaged among respondents with the disorder to obtain mean individual-level effect associated to it.

We used the population attributable risk proportion (PARP) to describe societal-level effects, which could be used to evaluate the expected effects on full role limitation of either preventing or successfully treating one or more of the disorders at the population level. PARPs were calculated in a similar way as the individual-level effects using the same two estimates: (a) the base estimate from the actual data and (b) the predicted value under the counterfactual assumption that the disorder in question has been removed from the population. These two estimates were averaged across the entire sample and the percentage difference between the two was computed. PARP can be interpreted as the proportion of days totally out of role that could have been alleviated if the predictor disorder did not exist. The same procedure was used to calculate individual and societal-level effects of the overall categories any physical disorder, any mental disorder, and any disorder. Given that models were adjusted by number of comorbid disorders and its interaction with the specific disorder, the different patterns of comorbidities were taken into account in the estimation of both individual and societal-level effects.

The SASH survey data are geographically clustered and weighted. Specific design-based methods were, therefore, needed to obtain accurate estimates of standard errors and statistical significance. The Taylor series linearization method implemented in SAS was used to do this for the basic model. The Jackknife Repeated Replication method implemented in a SAS macro specifically developed for this purpose was employed to estimate the standard errors of the individual-level and societal-level effects. Significance tests were consistently evaluated at *α* = 0.05, using two-sided design-based tests.

## Results

### Distribution of days out of role

Table 1 below shows the distribution of days out of role within the last 30 days prior to the interview for the entire sample. Overall 12.4 % of respondents reported any days out of role due to mental or physical disorder, and 10.8 % of them reported 21–30 days out of role due to any mental or physical disorder. The mean days out of role among those respondents with any days out of role was 7.9 and the median was 3.8.

**Table 1:** Distribution of days totally out of role in the month previous to the interview

	%	SE
Any days out of role	12.4	0.7
1 day	10.0	1.7
2 days	21.4	2.1
3–5 days	30.0	2.6
6–10 days	17.5	2.0
11–20 days	10.3	1.5
21–30 days	10.8	1.8
Median among respondents with positive days out of role	3.8	0.3

The SASH study

### Category of disorder prevalence and mean number of days totally out of role per year

The prevalence of categories of disorders assessed is presented in Table 2, together with mean yearly days out of role for each of the 10 disorder categories assessed. More than half of the sample (64.7 %) reported any (either mental or physical) disorder. The proportion of respondents reporting any physical disorders is substantially higher (more than three times higher) than those reporting any mental disorders (59.5 % with any physical disorder and 16.8 % reporting any mental disorder). With regard to specific mental disorders, 8.4 % reported anxiety disorders, 5.5 % reported depression and 5.7 % reported substance abuse. Those with anxiety disorders reported the highest mean days out of role (28.2 days). This was followed closely by those with depression, who reported 27.2 days out of role per year. The majority of respondents (63.0 %) who reported a disorder also reported another disorder. The average number of disorders reported by respondents reporting at least one disorder was 2.3.

**Table 2:** Prevalence of categories of disorders and mean annual days totally out of role (DOR)

Disorder	Prevalence (%)	SE %	Mean yearly DOR	SE mean
Depression	5.5	0.5	27.2	4.6
Any anxiety disorder	8.4	0.6	28.2	3.4
Any substance disorder	5.7	0.6	19.1	5.5
Headache or migraine	32.6	1.3	16.7	1.9
Arthritis	10.1	0.5	24.7	3.9
Pain	31.5	1.1	21.5	2.5
Cardiovascular	19.7	0.8	21.1	2.1
Respiratory	19.3	1.0	18.1	2.3
Diabetes	5.6	0.4	19.4	4.3
Digestive	5.5	0.5	17.9	4.6
Any mental	16.8	0.9	23.6	2.7
Any physical	59.5	1.2	15.5	1.4
Any disorder	64.7	1.1	15.3	1.3
Respondents with positive days out of role	–	–	96.2	6.5
All respondents	–	–	11.9	1.0
Respondents with positive days out of role (median)			46.1	3.9

The SASH study

The odds ratios (OR) evaluating comorbid conditions further reflect the substantial comorbidity in the sample. A positive OR (i.e. an OR >1) reflects elevated odds of a particular condition. In this study, almost 87 % of the ORs between pairs of disorders were positive and most of them were significantly >1 (73.3 %). The median of the ORs between pairs of physical disorders (physical–physical disorders) was 2.8 [interquartile range (IQR): 2.2–4.1]. It was found to be higher than that between physical and mental disorders (median = 1.56 and IQR: 1.0–2.4) as well as higher than between mental and mental disorders (median = 2.59 and IQR: 18–4.3). Table 3 presents the OR for pairs of individual disorders.

**Table 3:** Odds ratios for pairs of disorders

DX	Any anxiety disorder OR (95 % CI)	Any substance disorder OR (95 % CI)	Headache or migraine OR (95 % CI)	Arthritis OR (95 % CI)	Pain OR (95 % CI)	Cardiovascular OR (95 % CI)	Respiratory OR (95 % CI)	Diabetes OR (95 % CI)	Digestive OR (95 % CI)
Depression	4.2 (3.0, 6.0)	1.8 (1.0, 3.3)	1.9 (1.4, 2.6)	1.6 (1.0, 2.4)	2.2 (1.5, 3.2)	1.4 (1.1, 1.9)	1.8 (1.2, 2.7)	0.9 (0.5, 1.6)	2.0 (1.1, 3.5)
Any anxiety disorder		2.6 (1.7, 4.1)	2.3 (1.7, 3.0)	1.8 (1.3, 2.5)	2.2 (1.7, 2.8)	1.9 (1.4, 2.6)	2.1 (1.6, 2.8)	1.5 (1.0, 2.3)	2.4 (1.6, 3.5)
Any substance disorder			0.9 (0.6, 1.3)	0.8 (0.5, 1.4)	1.0 (0.7, 1.3)	0.6 (0.4, 0.9)	1.2 (0.8, 2.0)	0.5 (0.2, 1.3)	1.2 (0.7, 2.1)
Headache or migraine				2.4 (1.8, 3.1)	4.3 (3.4, 5.3)	2.6 (2.1, 3.2)	2.2 (1.8, 2.8)	1.4 (1.0, 2.2)	2.8 (2.1, 3.8)
Arthritis					8.2 (6.0, 11.2)	7.7 (5.8, 10.2)	2.7 (2.0, 3.6)	4.7 (3.3, 6.7)	3.1 (2.2, 4.4)
Pain						4.1 (3.4, 4.8)	3.2 (2.7, 4.0)	2.2 (1.6, 2.9)	4.0 (2.9, 5.6)
Cardiovascular							2.1 (1.8, 2.6)	11.8 (7.8, 17.9)	3.2 (2.4, 4.2)
Respiratory								1.4 (1.0, 1.9)	2.4 (1.7, 3.3)
Diabetes									1.7 (1.0, 2.9)

Table 4 presents the average individual-level effect (column 2) of each disorder on days out of role, obtained from simulations taking into account the comorbidity patterns of the representative sample. Overall, anxiety disorders had the highest individual-level effect (12.7, SE = 3.4), meaning that, in comparison with not having them, an average of 12.7 net additional days totally out of role per year were associated with anxiety disorders. Pain disorders had the second largest value (9.9 mean additional days, SE = 2.9). The mean individual effect associated with depression was also high (10.1 days), although it was not statistically significant (SE = 5.7).

**Table 4:** Additional yearly days totally out of role (‘individual effects’) and PARPs (“societal effects”) for each disorder

	Additional days totally out of role		Population attributable risk proportion (PARP)	
	Mean	SE	%	SE (%)
All disorders	8.9	2.0	46.8	10.6
All mental	11.9	2.5	16.1	3.4
All physical	7.7	2.1	37.6	10.5
Depression	10.1	5.7	4.8	2.7
Any anxiety disorder	12.7	3.4	9.0	2.5
Any substance disorder	8.2	5.7	3.3	2.3
Headache or migraine	3.1	2.3	8.5	6.3
Arthritis	1.7	4.4	1.4	3.8
Pain	9.9	2.9	25.8	6.7
Cardiovascular	2.2	3.6	3.8	6.3
Respiratory	3.9	2.5	6.2	4.0
Diabetes	1.8	4.4	0.8	2.0
Digestive	−1.3	5.0	−0.7	2.4

Additional to those estimated for the average individual without the disorder

Societal effects expressed as PARPs are also presented in Table 4 (column 4). In general, the estimated PARP was 46.8 % when all disorders were considered at once, meaning that within the theoretical assumption that all these disorders could be prevented, the total number of days totally out of role would be reduced by 46.8 %. Physical disorders had the highest impact at the society level, accounting for 37 % of the days totally out of role, compared to only 16.1 % accounted for mental disorders. Among the mental disorders, anxiety disorders had the highest PARP in relation to days out of role (9.0 %) followed by depression (4.8 %) and substance disorder (3.3. %). When focusing on individual physical disorders, pain is the one with the highest societal effect (PARP = 25.8 %, SE = 6.7).

## Discussion

The findings from this study indicate that both physical and mental disorders are associated with substantial days out of role in South Africa with physical disorders having higher PARP than mental disorders. The SASH data also show that there is substantial comorbidity of mental and physical disorders in South Africa, consistent with prior international and local findings [12].

The finding that 12.4 % of respondents experienced any days out of role is comparable to data from other countries in the WMHS [12] (12.8 % for all the other countries). We found that almost two-thirds of the sample (64.7 %) reported any (either mental or physical) disorder with 59.5 % reporting any physical disorder and 16.8 % reporting any mental disorder, and this is comparable with other countries. As in other countries, the proportion of respondents reporting physical disorders was substantially higher (more than four times higher) than those reporting solely mental disorders, with previous work locally and elsewhere indicating that pain disorder has the highest associated PARP [12]. The findings are also consistent with previous local and international work which has found that the presence of enduring depression or anxiety disorders is associated with substantial reduction in earnings in both employed and unemployed South Africans [27].

Accounting for the effects of comorbidity significantly decreases almost all disorder-specific effects and thus allows for more accurate estimates of the precise effects of disorders on disability [12]. It is important to note that average disorder-specific individual effects vary depending on whether the individual has only that particular disorder or it is comorbid with other disorders (specifically, it depends on the number of comorbidities and which disorders are involved). The estimate presented in Table 4 takes into account the pattern of comorbidities observed in a representative sample of the adult general population.

With regard to the public health implications of our study, there are several suggestions. First, the disorder combinations with the strongest associations (i.e. highest OR’s from Table 3) and the largest PARPs (from Table 4) are those most important priorities for intervention or prevention. Second, we should consider health system improvements that facilitate treatment of comorbid conditions. For example, research reported in the Lancet South Africa series in 2009 [28] emphasized that South Africa faces concurrent epidemics including HIV/AIDS, tuberculosis, mental disorders and chronic diseases of lifestyle. Comorbid conditions, therefore, require integrated models of care for health and economic reasons [28]. In a recent systematic review and meta-analysis, van der Feltz-Cornelis and colleagues explored the efficacy of combining treatment for depression and diabetes. Their meta-analysis of 14 randomized controlled trials shows a reduction of depressive symptoms when psychotherapeutic intervention is combined with self-management of diabetes [29]. There has already been evidence of mental health care integrated in some of South Africa’s HIV sites. This includes targeted mental health care for people living with HIV and training to HIV service providers to detect common mental disorders [30].

The findings that both mental and physical disorders can impede functioning as measured by productive employment and participation in other social roles, have several implications for policy. First, there is a need for early interventions in integrated health services to address multiple comorbid conditions, including the growing burden of mental disorders. Secondly, provision of disability grants for people with physical or mental disorder is essential to provide financial protection against the disabling consequences of mental and physical illness [31].

We note that there are limitations to this analysis. First, the prevalence of categories of physical disorders is based on self- report. However, methodological studies in both the USA and the United Kingdom have documented good concordance between self-report of these conditions and corresponding medical records [17, 21]. Second, data were drawn from a cross-sectional study which gives a sense of the strength of the relationship between mental and physical disorders and days out of role as well as the odds of comorbid conditions, but does not allow causality to be determined. We note also that although HIV/AIDS status was included in the questionnaire, few respondents were willing to admit that they were HIV positive. Therefore, HIV status has not been included in this analysis. Given the high prevalence of HIV in South Africa, and the physical and social consequences of the illness, days out of role could well be a consequence of HIV/AIDS. As noted in previous publications from the SASH dataset, psychosis, dementia and child and adolescent mental disorders have also not been included in this analysis.

## Conclusion

This paper provides new information about the potential impact of mental and physical disorders on sustainable, productive employment and other important daily roles. Furthermore, the paper highlights policy directives regarding the importance of providing economic protection for health related disability, improving prevention and treatment strategies through early prevention and integrated care, and including mental health in all basic packages of healthcare.

## Footnotes

1 ‘Coloured’ is a racial category in South Africa. The category refers to people of mixed race descent.
